# Barcoding Quantitative PCR Assay to Distinguish Between *Aedes aegypti* and *Aedes sierrensis*

**DOI:** 10.3390/tropicalmed10080230

**Published:** 2025-08-15

**Authors:** Miguel Barretto, Annika Olson, Dereje Alemayehu, Ryan Clausnitzer, Eric J. Haas-Stapleton

**Affiliations:** Alameda County Mosquito Abatement District, Hayward, CA 94545, USA; miguelb@mosquitoes.org (M.B.); aolson@contracostamosquito.com (A.O.); dereje@mosquitoes.org (D.A.); ryan@mosquitoes.org (R.C.)

**Keywords:** molecular identification, *Aedes aegypti*, *Aedes sierrensis*, mosquito control

## Abstract

The accurate identification of mosquito species is critical for effective mosquito surveillance and control, especially when presented with morphologically similar species like *Aedes aegypti* and *Aedes sierrensis*. Damaged specimens and morphologically similar life stages such as eggs and larvae make it difficult to distinguish *Aedes aegypti* from *Aedes sierrensis* using microscopy and taxonomic keys. To address this, the *AegySierr.*ID-qPCR assay, a multiplex quantitative PCR assay that utilizes single-nucleotide polymorphisms within the *mitochondrial cytochrome oxidase subunit I* gene, was developed to distinguish between these two species. The assay was tested on DNA extracted from the eggs, larvae, and adults of both species, as well as from environmental DNA (eDNA) collected from natural mosquito reproduction sites. It demonstrated a high diagnostic accuracy across multiple life stages, with a sensitivity exceeding 95% for most groups and specificity exceeding 90%, except for field-collected adult *Ae. sierrensis* (75%). For eDNA samples, the assay achieved 100% sensitivity and 94% specificity for samples classified as *Ae. sierrensis* and 91% sensitivity and 86% specificity for *Ae. aegypti*. A two-graph receiver operating characteristic analysis was also used as an alternate method with which to establish Ct thresholds for interpreting results from unknown samples. The *AegySierr.*ID-qPCR assay enables the rapid and sensitive identification of *Ae. aegypti* and *Ae. sierrensis* from specimens and eDNA, and may be of use in mosquito surveillance programs.

## 1. Introduction

As *Aedes* (*Stegomyia*) *aegypti* (Linnaeus) mosquitoes continue their spread in North America, the risk of mosquito-borne diseases such as chikungunya, dengue, and Zika may increase [1,2,3]. These diseases are of public health concern because they can cause fever, joint pain, and, in severe cases, neurological complications, and adverse fetal outcomes in affected individuals [3,4,5,6]. When *Aedes aegypti* becomes established in a community, they discourage outdoor activity and reduce economic productivity by increasing nuisance biting, disease transmission risk, and public health burdens [7].

*Aedes aegypti* reproduces principally in water-filled containers that occur around people’s homes [8,9]. *Aedes* (*Ochlerotatus*) *sierrensis* (Ludlow) is broadly distributed in the western United States [10], transmits *Dirofilaria immitis* (heartworm) to dogs [11], and reproduces primarily in tree cavities when they contain water [12]. Oviposition traps, containers filled with water and a substrate that passively collect mosquito eggs for monitoring and control, may capture eggs from both *Ae. aegypti* and *Ae. sierrensis* [13]. However, the immature stages of *Ae. sierrensis*, the eggs and larvae, are morphologically similar to those of *Ae. aegypti* [8,14,15]. Determining where these mosquitoes propagate is crucial for effective control, and the accurate identification of vector species is essential, as different approaches may be required for managing native versus non-native mosquitoes [16]. Mosquito control programs may emphasize containment and eradication for non-native species such as *Ae. aegypti* [17], while strategies for native species such as *Ae. sierrensis* may focus on habitat management and nuisance reduction [18]. Early identification at immature life stages may allow for a timelier implementation of the appropriate control measures to prevent further spread.

Traditional morphological taxonomy, widely used to identify mosquito larvae and adults, has several limitations. Accurate identification requires a trained entomologist familiar with binomial keys, and the process is time-consuming and challenging when specimens are damaged and lack key distinguishing features [19,20]. Although species can be differentiated by eggshell morphology using scanning electron microscopy, such imaging techniques are costly and may not be available to most vector control laboratories [21,22]. Additionally, *Aedes* eggshells generally lack sufficient distinguishing characteristics to visually identify them as specific species [23]. When morphological taxonomy fails to produce a definitive result, an alternative method is needed for accurate species identification.

Molecular approaches such as loop-mediated isothermal amplification (LAMP) and DNA sequencing have been explored for species identification [24]. LAMP enables rapid DNA amplification and can be interpreted visually, but its reliance on up to six primers and a complex primer design increases the risk of non-specific amplification and complicates assay development. DNA sequencing provides high-resolution species identification; yet, it typically requires expensive instrumentation, post-amplification processing, and bioinformatics expertise, factors that may limit its feasibility for routine use in vector surveillance programs. In contrast, quantitative PCR (qPCR) offers a practical balance of sensitivity, specificity, and scalability, making it especially suitable for vector control laboratories that often already use qPCR to test mosquitoes for arboviruses such as West Nile virus [25].

Molecular genetic approaches, such as DNA barcoding, can supplement morphological taxonomy for species identification [26,27]. The mitochondrial Cytochrome Oxidase subunit I (COI) barcoding region is particularly useful for identifying species within the *Aedes* genus, as it contains conserved regions within species and single-nucleotide polymorphisms (SNPs) that differentiate among species [28,29]. Using SNPs within the *COI* gene, we developed a multiplex qPCR assay designed to distinguish *Ae. aegypti* from *Ae*. *sierrensis* (*AegySierr.*ID-qPCR). In addition to identifying physical specimens, the *AegySierr.*ID-qPCR assay was evaluated for use with environmental DNA (eDNA) extracted from water samples collected at mosquito reproduction sites, including artificial containers and natural habitats such as phytotelmata [30,31]. This study aimed to evaluate the diagnostic performance of the assay across multiple life stages of *Ae. aegypti* and *Ae. sierrensis*, as well as for eDNA from water samples collected during mosquito surveillance. The assay was developed with a focus on western North America, where the geographic ranges of *Ae. aegypti* and *Ae. sierrensis* may overlap, and distinguishing between them may be important for effective vector control.

## 2. Materials and Methods

### 2.1. Mosquito Collection

*Aedes aegypti* were originally collected from Mission Viejo in Orange County, California (USA; strain *Ae. aegy*-MV) using encephalitis vector survey traps that were baited with dry ice (EVS traps; catalog number 2801A, BioQuip, Rancho Dominguez, CA, USA) and reared in an insectary under standard growth conditions. Laboratory-reared F1-generation *Ae. aegypti* adults and F2-generation eggs and third instar larvae were collected and frozen at −80 °C until use. A laboratory strain of *Ae. sierrensis*, originally derived from a population in Cotati (Sonoma County, CA, USA), was used to obtain tissue samples from all life stages of the species (strain *Ae. sierr*-C). *Ae. sierr*-C larval samples were stored in 70% ethanol and adults frozen at −20 °C prior to use. Additional larval and adult stages of *Ae. sierrensis* were field-collected from Alameda County in Oakland (CA, USA) using EVS traps for adult stages and hand-collected with a larval sampling cup for the immature stages (BioQuip, Rancho Dominguez, CA, USA). Adult and larval mosquitoes were identified to species using a Nikon SMZ1000 dissection microscope (Nikon, Tokyo, Japan), chill table (catalog number 1431, BioQuip, Rancho Dominguez, CA, USA), and binomial key [32]. Adult or larval mosquitoes or eggs were individually placed in a 2 mL microcentrifuge bead mill tube that contained a 5 mm glass bead (Fisher Scientific, Waltham, MA, USA) and stored at −80 °C until use (Table 1).

### 2.2. eDNA Sampling

Water samples were collected from natural and artificial mosquito larval habitats, including treeholes, artificial containers, 1-gallon oviposition traps [33], and In2Care traps (In2Care BV, Wageningen, The Netherlands). Water was removed from containers using a handheld aspirator cleaned before each use by immersion in 10% bleach, followed by three rinses with tap water. For each collection site, three 50 mL conical vials were filled with 40 mL of water, placed on ice for transport, and, subsequently, stored at −20 °C until filtration. Water samples were collected from treeholes and oviposition traps, with and without observable larvae, across multiple counties in California (USA) [33].

### 2.3. eDNA Filtering

Water samples were filtered as described previously [34] through a 10 µm nylon prefilter using a 47 mm filter holder to remove large particulates (MilliporeSigma, Burlington, MA, USA; Cole-Parmer, Vernon Hills, IL, USA). eDNA was captured on polyethersulfone (PES) membrane filters (0.22 µm pore; Cobetter North America Corporation, Waltham, MA, USA) using a vacuum filtration apparatus (Fristaden Lab, Reno, NV, USA). This pore size and filter media were chosen to maximize capture efficiency for species-specific eDNA, as shown in [35]; 0.22 µm PES capsules recovered more total eDNA and yielded lower qPCR cycle threshold (Ct) values than cellulose-nitrate, polycarbonate track-etched filters, glass-fiber filters, and ethanol precipitation. Each sample was processed in triplicate by filtering 25 mL aliquots through separate PES filters. The vacuum filtration apparatus was decontaminated between samples by sequentially soaking in 10% bleach for 10 min, washing with 10% Alconox detergent (Alconox, White Plains, NY, USA), rinsing three times with deionized water, and drying with 200-proof ethanol (Fisher Scientific, Waltham, MA, USA). After filtration, each PES filter was cut into approximately 3 mm slices to maximize exposed retentate surface area and placed into sterile 2 mL centrifuge tubes that contained 0.5 g of 425–600 µm acid-washed glass beads (MilliporeSigma, Burlington, MA, USA), and subsequently stored at −20 °C until extraction.

### 2.4. Alternative Prefilter Media: Coffee Filters

Coffee filters (Trader Joe’s, Monrovia, CA, USA) were evaluated as an alternative prefilter because they have a pore size similar to the nylon prefilter (10–30 µm [36]) and are less costly. Water samples were gravity-filtered through a coffee filter placed inside a funnel until flow was impeded, at which point the filter was replaced to complete the prefiltration. In subsequent tests, coffee filters were cut to a 5 cm diameter using a disc punch (UCEC-TECH, Dongguan, China) to standardize the filtration area. All filtration steps using coffee filters followed the same decontamination and storage procedures as described above for nylon prefilters. Eight field-collected samples were split and filtered in triplicate for each type of filter. DNA was extracted from each filtered replicate and analyzed in triplicate using qPCR. Ct values from these samples were used to evaluate differences between the prefilter media. Statistical software (Graphpad Prism, version 10.5.0, Dotmatics: Moston, MA, USA, 2025) was used to perform unpaired *t*-tests with Welch’s correction and a two-tailed *p* value was used to assess statistical significance.

### 2.5. DNA Extraction

Ethanol was removed from mosquito specimens using a pipette and dried 15 min before extracting the DNA. Genomic DNA was isolated from all mosquito life stages using a MagMAX-96 Viral RNA Isolation Kit (Thermo Fisher Scientific, Waltham, MA, USA), which isolates both DNA and RNA, using a Kingfisher Duo Prime Purification System (Thermo Fisher Scientific, Waltham, MA, USA) according to manufacturer’s instructions. The concentration of the eluted DNA was quantified using a Nanodrop 2000 spectrophotometer (Thermo Fisher Scientific, Waltham, MA, USA). Samples containing eDNA were extracted from filters as described previously [37]. The elution volume for all tissue and eDNA samples was 50 µL, using MagMAX-96 Elution Buffer for the former and Buffer AE for the latter.

### 2.6. qPCR Barcoding Assay for Identifying Ae. sierrensis and Ae. aegypti

**Figure 1:**
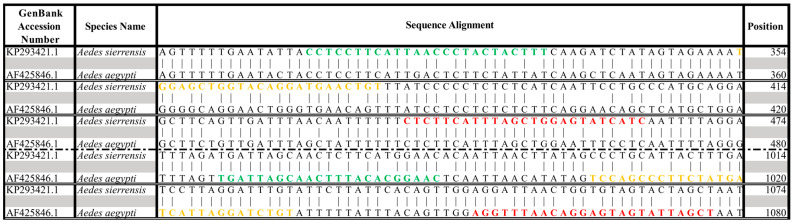
Multiple sequence alignment for *Ae. aegypti* and *Ae. sierrensis*. Forward primers are colored in green, probes in yellow, and reverse primers in red.

The *AegySierr.*ID-qPCR assay was designed and validated for binary species discrimination between *Ae. aegypti* and *Ae. sierrensis*, and its use is intended for regions where these two species co-occur. Assay performance in the presence of other sympatric *Aedes* species was not evaluated in this study. The *AegySierr.*ID-qPCR primer and probe sequences that targeted the COI region of *Ae. aegypti* and *Ae. sierrensis* (GenBank No. AF425846.1 and KP293424.1, respectively) were designed using Primerquest software (Table 2; version 2.2.3; Integrated DNA Technologies, Coralville, IA, USA). MUSCLE multiple sequence alignment tool [38] was utilized to align DNA sequences and visualize the location of SNPs (Figure 1). The *Ae. aegypti* probe (*aegypti*-PRB) was labeled with ABY and Iowa Black RQ (IAbRQSp) quencher at the 3′-end and the *Ae. sierrensis* probe (*sierr*-PRB) with FAM and IowaBlack FQ (IABkFQ) quencher at the 3′-end (Integrated DNA Technologies, Coralville, IA, USA). The Primetime Gene Expression Master Mix (Integrated DNA Technologies, Coralville, IA, USA) was prepared according to the manufacturer’s recommendations, using 5 μL of template DNA (10.5–2790 ng), 1.8 µL of each primer with a final reaction concentration of 900 nM, 0.5 µL of each probe with a final reaction concentration of 250 nM, 10 μL of the Master Mix, and nuclease-free water for a final volume of 20 μL. The reaction mixture was dispensed into a MicroAmp Optical 96-Well Reaction Plate (Thermo Fisher Scientific, Waltham, MA, USA), which was vortexed for 15 s at maximum speed and, subsequently, centrifuged for 15 s using an MPS 1000 Mini PCR Plate Spinner (Labnet International, Inc., Edison, NJ, USA). Quantitative PCR was performed on a QuantStudio 5 Real-Time PCR System (Thermo Fisher Scientific, Waltham, MA, USA) with the following cycling conditions: 95 °C for 3 min, followed by 40 cycles of 95 °C for 5 s and 63.4 °C for 30 s. Baseline and threshold values were automatically determined by the QuantStudio Design and Analysis Software v2.6 (Thermo Fisher Scientific, Waltham, MA, USA).

### 2.7. Species Identification, Sensitivity, and Specificity Using the AegySierr.ID-qPCR Assay

For tissue samples, true positives were defined as specimens that were taxonomically verified by microscopy using a binomial key and that showed amplification with correct species-specific probe in the *AegySierr.*ID-qPCR assay. True negatives were defined as samples that showed no amplification with probes targeting non-matching species. False positives were cases in which non-specific amplification occurred with a probe not corresponding to the verified species identity, while false negatives were samples that failed to amplify the target sequence despite prior taxonomic verification.

The sensitivity and specificity measures of the *AegySierr.*ID-qPCR assay were calculated using Excel (Microsoft Corporation, Redmond, WA, USA) for each sampling region, species, and location with the following equations [39]:

$$Sensitivity=\frac{True\;Positive}{True\;Positive+False\;Negative}\;Specificity=\frac{True\;Negative}{True\;Negative+False\;Positive}$$

Confidence intervals (CIs) were calculated using the simple asymptotic method without continuity correction, and any upper bound of the confidence intervals over 100% were rounded down to 100% [40]:

$$CI=p\pm z\sqrt{\frac{p(1-p)}{n}}$$

where *p* is the diagnostic method proportion and *z* equals 1.96 for 95% confidence.

Unlike tissue samples, which could be independently verified through morphological identification, eDNA samples lacked visual species confirmation. To estimate classification performance when verified labels were unavailable, Latent Class Analysis (LCA) was used to infer the likely class of each sample based on the amplification pattern across qPCR triplicates [41]. Replicate qPCR results were first converted into two-category variables for each probe target, with amplification denoted as 2 and no amplification as 1. Results were then organized into six variables per site sample, reflecting three replicates for each of the two species-specific probes: *sierr*-PRB and *aegypti*-PRB. Latent class models were implemented using the poLCA package in R to infer underlying categories in the qPCR results [42].

Ct values were reported as the mean ± standard deviation (SD). For comparison between sample types, combined mean Ct values and combined SD were calculated with each regional subgroup pooled life stage and region (Supplementary File S1 shows the equations used to consolidate multiple means and SD values) [43].

To establish Ct cutoffs with unknown tissue specimens in cases where both probes amplify, two-graph receiver operating characteristic (TG-ROC) analysis was used as an alternative to assuming that any dual-probe amplification indicates the presence of both species in the sample [44]. Diagnostic measures were calculated by evaluating maximum Ct thresholds in 0.5-cycle increments. For each tissue sample type, if the sensitivity and specificity curves intersected, the Ct value at the point of intersection was selected as the cutoff. In cases where no distinct intersection was observed, a default cutoff of Ct = 40 was applied because this corresponded to the maximum number of qPCR cycles used in the *AegySierr.*ID-qPCR assay.

## 3. Results and Discussion

### 3.1. DNA Concentrations and Ct Values

*Ae. sierrensis* and *Ae. aegypti* display similar oviposition behaviors, produce eggs with similar morphological characteristics [10,45,46], and share a 89% nucleotide identity in the *COI* gene (accession numbers KP293421.1 and AF425846.1 [47]). Although dissimilar nucleotides may be sufficient for differentiating between the two species, factors such as melting temperature differences and secondary structure formation in nucleic acids can limit the regions available for developing a diagnostic PCR assay [48,49].

The *AegySierr.*ID-qPCR assay used the same region of the COI gene and was tested on samples collected from several geographic regions and containers to assess how different mosquito populations responded. Populations from different sampling sites were expected to exhibit both genetic variability and differences in DNA content that may have been influenced by factors such as the body size and sample preservation method. To account for these variables, DNA concentrations were measured for all tissue samples. The concentration of DNA that was extracted from mosquitoes ranged from 2.1–125 ng/µL for an egg (mean ± SD: 13.6 ± 9.9 ng/µL), 23.1 to 370 ng/µL for a larva (128.7 ± 72.0 ng/µL), and 11.8–78.2 ng/µL for an adult (95.5 ± 91.6 ng/µL).

**Figure 2:**
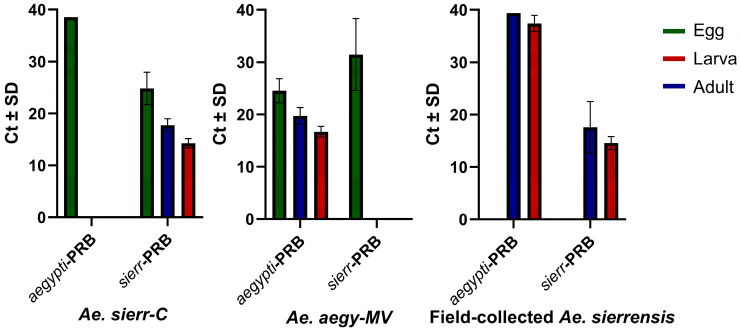
Mean Ct values for *aegypti*-PRB and *sierr*-PRB probes for egg (n = 119), larva (n = 31), and adult (n = 72) stages of *Ae. aegypti* collected from Orange County, and *Ae. sierrensis* collected from Alameda and Sonoma counties. Error bars represent standard deviations.

Differences in DNA concentrations between Alameda County *Ae. sierrensis* and *Ae. sierr*-C samples may reflect differences in sample collection and storage methods. *Ae. sierr*-C larvae were ethanol-preserved and originated from a laboratory-reared generation, while Alameda County *Ae. sierrensis* adults and larvae were stored at −80 °C without ethanol. The absence of ethanol may have contributed to the lower DNA concentrations observed in Alameda adult samples relative to larvae [50]. Additionally, the lowest DNA concentration observed across all egg samples (2.1 ng/µL) yielded successful amplification in the *AegySierr.*ID-qPCR assay. Overall, Ct values followed an expected trend across life stages: the lowest in adults, intermediate in larvae, and the highest in eggs (Figure 2).

Because Ct values serve as indicators for the gene copy number, lower Ct values reflect a higher quantity of the target gene in the sample [51]. Therefore, it was anticipated that the *Ae. sierr*-C and *Ae. aegy*-MV eggs would have the highest average Ct values, followed by adults and larvae. The *AegySierr.*ID-qPCR assay was developed using mitochondrial DNA sequences and the number of mitochondria can vary across life stages with the resulting differences in the mitochondrial genome copy number potentially affecting Ct values [52]. Unlike tissue samples, where the target organism makes up a dominant proportion of DNA content, eDNA samples contain a mixture of nucleic acids from all organisms present in the water at the time of sampling. As a result, the total DNA concentration does not reflect the abundance of mosquito DNA specifically and may be overrepresented by bacterial, non-target insect, or other DNA. Therefore, the DNA concentration was not quantified for eDNA samples, as it may not accurately represent the amount of mosquito-derived DNA.

### 3.2. eDNA Sample Species-Designation by Latent Class Analysis

Models with two, three, and four classes were evaluated, with the three-class model selected based on optimal fit across multiple criteria: Akaike Information Criterion (AIC = 1009.738), Bayesian Information Criterion (BIC = 1072.218), Likelihood Ratio (G^2^ = 40.048), and Chi-square goodness-of-fit (Χ^2^ = 41.096) [41,42]. These values were the lowest among the tested models, supporting the three-class model as the best fit for the data.

**Figure 3:**
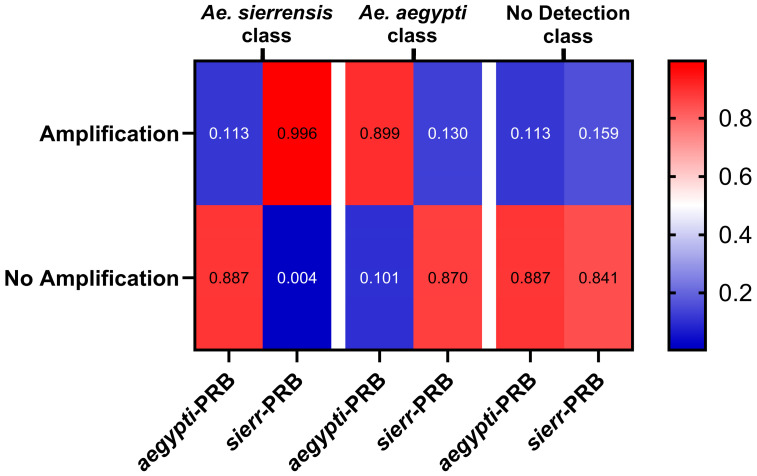
Amplification probabilities by probe and inferred class.

The latent classes were interpreted based on the response probabilities associated with each probe. One class showed a high probability of amplification for the *sierr*-PRB probe and a low probability with *aegypti*-PRB, indicating the presence of *Ae. sierrensis* in the sample. A second class showed the opposite pattern with a high amplification probability with *aegypti*-PRB and low with *sierr*-PRB. The third class showed low amplification for both probes, consistent with no detection of either species. These classes were interpreted as *Ae. sierrensis*, *Ae. aegypti*, and No Detection (Figure 3). The LCA-derived class for each sample was then used as a proxy truth value to evaluate the accuracy of the *AegySierr.*ID-qPCR assay with eDNA.

Because eDNA samples originate from trace quantities of DNA shed into the environment, they typically produced higher Ct values and showed greater variability in amplification compared to tissue-derived samples. This shift in Ct distributions limited the utility of a fixed Ct threshold to define positive detections. This combination of presumed low target DNA quantity and higher Ct variability limited the reliability of applying a fixed Ct threshold to define positive detections in eDNA. To address this, a simplified classification approach was used for eDNA samples, in which the presence or absence of amplification served as the primary diagnostic criterion. When both species-specific probes were amplified within a sample, the probe with the lower Ct value, which indicated a relatively higher target DNA abundance, was used to assign the species-level result. The sensitivity and specificity were then calculated using the same method applied to tissue samples, based on the agreement between the LCA-inferred class and the qPCR result for each extraction replicate. The same simple asymptotic method described above was used to calculate 95% CI.

### 3.3. AegySierr.ID-qPCR Assay Amplification Plots

**Figure 4:**
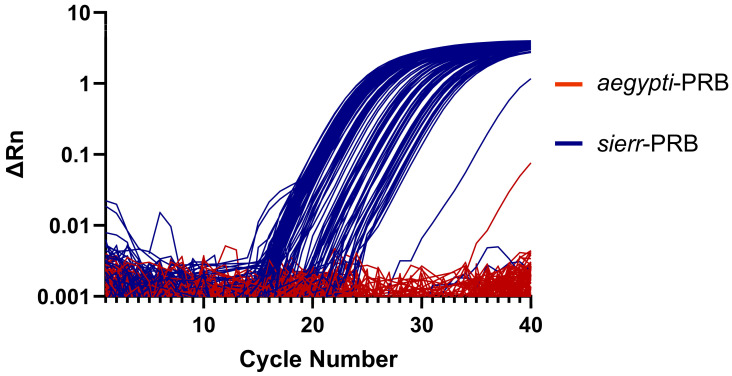
Amplification plots for each *Ae. sierrensis* egg sample collected from Sonoma County (n = 114), showing results for both *sierr*-PRB (blue) and *aegypti*-PRB (red) probes.

**Figure 5:**
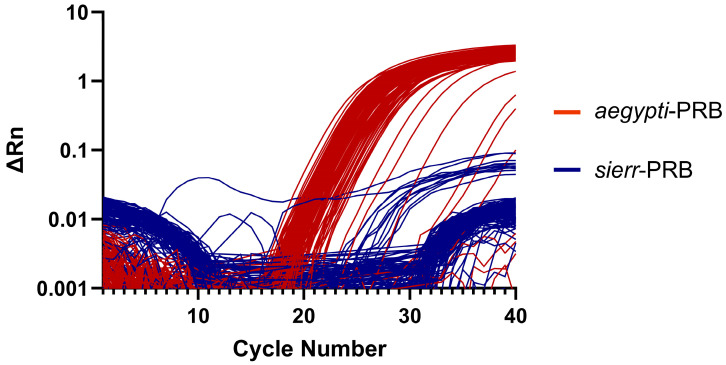
Amplification plots for each *Ae. aegypti* egg sample collected from Orange County (n = 119), showing results for both *sierr*-PRB (blue) and *aegypti*-PRB (red) probes.

The rate of false-positive amplification was low, occurring in fewer than 1% of *Ae. sierrensis* egg samples and 10% of *Ae. aegypti* egg samples, as indicated by the presence of amplification curves (Figure 4 and Figure 5) and Ct values (Table 3). However, there was a single instance of a false positive in which a verified *Ae. aegypti* egg sample from Orange County amplified the *sierr*-PRB probe and produced a lower Ct than the *aegypti*-PRB probe. Although this may suggest a low assay specificity for this specimen, the amplification curve was irregular and displayed a sharp increase in fluorescence during the early amplification phase (cycle number 3–13), which declined shortly after (Figure S1). Atypical amplification curves such as this may occur if the DNA template concentrations are too high [53]. In all other cases, when an incorrect probe was amplified, the Ct value that was produced from the amplification of the correct probe was lower, indicating the true positive.

The greatest standard deviation in Ct values was observed in *Ae. sierrensis* larval samples from Alameda County (Table 4), indicating a greater variation among larvae compared to the other life stages. Larval *Ae. sierrensis* also exhibited the highest false negative rate at 11.8% (Table 3). This increased variability may reflect both greater genetic diversity, and differences in body size and DNA concentration between field-collected samples and colony-reared *Ae. sierr*-C mosquitoes. The standard deviation of Ct values was lower in colony-reared *Ae. sierrensis* compared to field-collected *Ae. sierrensis* (Figures S2–S4 and Table 4).

For adult samples, *Ae. aegy*-MV had fewer false positives compared to adult *Ae. sierrensis*, but, when false positives occurred, the Ct values were closer to the Ct range observed for true positives (Figure S5). Given that the *AegySierr.*ID-qPCR assay is a multiplex qPCR assay that is capable of amplifying multiple targets simultaneously, occasional non-specific cross-amplification may occur. However, because *Ae. aegypti* and *Ae. sierrensis* typically occupy different ecological niches (artificial containers and treeholes, respectively), we expected only one species in each sample. Therefore, the observed false positives may have resulted from the non-specific amplification of the COI gene.

Although the *AegySierr.*ID-qPCR assay was not tested on pupal specimens, the genomic DNA present in pupae is expected to be identical to the DNA found in eggs, larvae, and adults. The quantity of genomic DNA isolated from *Ae. aegypti* pupae fall between that of larvae and adults [54]. Given the consistent genomic content across mosquito life stages and the comparable DNA concentrations, the *AegySierr.*ID-qPCR assay is likely to provide reliable species-level identification from pupal tissues, similar to its performance with egg, larval, and adult samples.

### 3.4. Comparison of Prefiltration Materials for eDNA Recovery

Paper coffee filters were compared to the 47 mm pre-cut nylon filters to determine if this less-expensive media was a cost-effective alternate prefiltration material for collecting eDNA. There was no significant difference between the Ct values between identical samples that were prefiltered using either coffee filters or nylon membrane filters (coffee: 31.18 ± 3.91 and nylon: 30.88 ± 3.712; mean difference = −0.30 ± 1.18; 95% CI: −2.68 to 2.08; *p* = 0.7996). An unpaired *t*-test with Welch correction revealed no statistically significant difference between groups (mean difference = −0.30 ± 1.18; 95% CI: −2.68 to 2.08; *p* = 0.7996). These results indicate that coffee and nylon prefilters were equally effective for removing coarse debris in eDNA samples without affecting downstream qPCR sensitivity. Because coffee filters are readily available, relatively inexpensive, and easy to use in the field, they offer a convenient alternative for prefiltration, especially in resource-limited or high-throughput monitoring settings. Given their comparable performance, samples were prefiltered using coffee or nylon filters.

### 3.5. eDNA Samples

**Figure 6:**
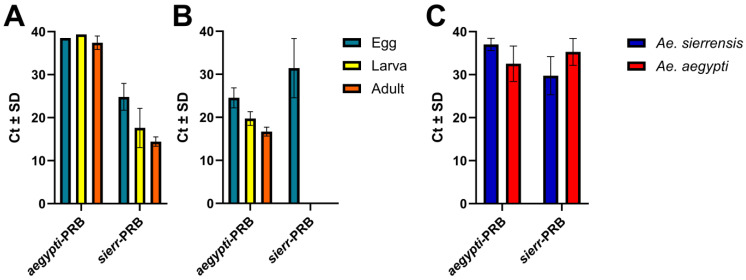
Mean Ct values ± standard deviation obtained from the *AegySierr.*ID-qPCR assay using species-specific probes (*aegypti*-PRB and *sierr*-PRB) for *Aedes sierrensis* (**A**) and *Ae. aegypti* (**B**) across three sample types that were previously identified with specific species using microscopy and binomial keys: egg, larva, and adult. Mean Ct values ± standard deviation for eDNA (**C**) for both species with *Ae. sierrensis*-designated samples and *Ae.aegypti*-designated samples.

Although 25 mL of water was assayed for each treehole or artificial container and the presence or absence of larvae recorded, the quantity of larvae was not enumerated. As a result, the concentration of mosquito DNA in each sample may have varied due to differences in larval density, mosquito life stage, or eDNA degradation before collection [55], resulting in a wider range of Ct values and greater variability in amplification plot curves (Figure 6, Figures S6 and S7). Similar to the atypical amplification previously described (Figure S1), one eDNA sample that was classified by the model as No Detection exhibited an amplification curve with a rapid increase in fluorescence during the early phase, followed by a sharp decline (Ct = 9.672).

Although environmental samples may contain DNA from a variety of organisms, the high specificity of probe-based qPCR limits off-target amplification. Nonetheless, non-target DNA and potential inhibitors present in natural breeding sites could interfere with amplification efficiency, particularly when the target DNA is low.

### 3.6. Sensitivity and Specificity of the AegySierr.ID-qPCR Assay

**Figure 7:**
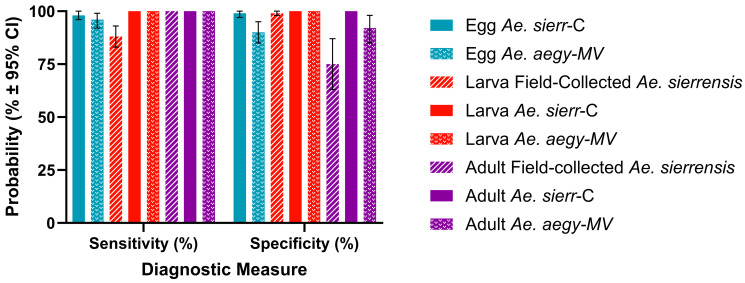
Diagnostic sensitivity and specificity of the qPCR assay with error bars representing 95% confidence intervals calculated using the simple asymptotic method without continuity correction.

Assay sensitivity reflects the ability of the assay to correctly identify samples containing the target species, while specificity indicates the ability to correctly exclude non-target species [39]. For the *AegySierr.*ID-qPCR assay, both metrics assess how effectively the assay distinguishes between species based on SNPs within the COI gene. The *AegySierr.*ID-qPCR assay demonstrated high sensitivity (Figure 7), with values exceeding 95% for all species and life stages except for *Ae. sierrensis* larvae from Alameda County (88%). High specificity was also observed, with most samples scoring above 90% (Figure 7). An exception was the field-collected adult *Ae. sierrensis* mosquitoes which produced a specificity of 75% (Figure 7). The greater genetic heterogeneity for field-collected mosquitoes in this group may have led to inefficient or non-specific primer or probe annealing to the nucleic acid during qPCR.

**Figure 8:**
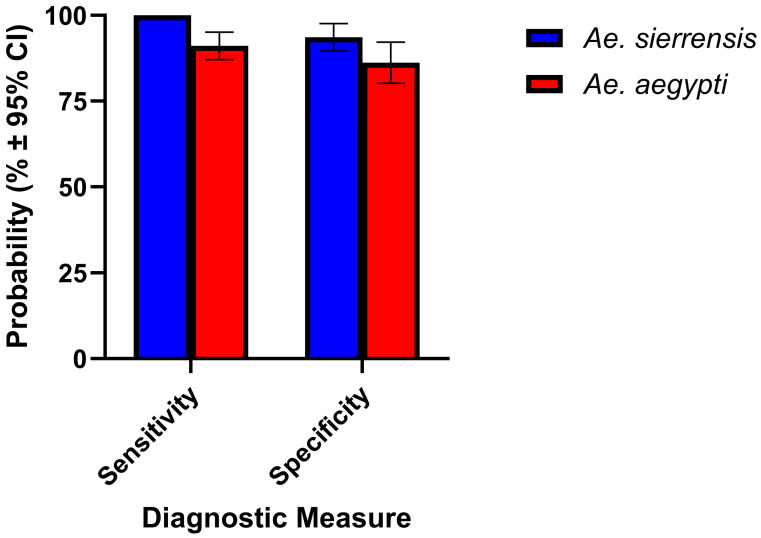
Accuracy and precision of the *AegySierr.*ID-qPCR assay according to diagnostic measures in eDNA by LCA-designated classes. Error bars represent ± 95% confidence intervals.

Species identification from field-collected eDNA samples was reliably achieved with the *AegySierr.*ID-qPCR assay, as indicated by diagnostic performance metrics derived from LCA (Figure 8). For samples classified as *Ae. sierrensis*, the assay achieved a sensitivity of 100% and a specificity of 94% (Figure 7). For *Ae. aegypti*-designated samples, or samples that had aa high probability of being in the *Ae. aegypti* class by LCA, the sensitivity was 91% and specificity was 86% (Figure 8). Confirmed true positive amplifications showed lower Ct values, with the *sierr*-PRB probe producing a mean Ct of 30.090 ± 4.482 and the *aegypti*-PRB probe a mean Ct of 32.535 ± 4.126 (Figure 6). Notably, eDNA samples had a higher sensitivity than the field-collected larva samples from Alameda County. This may be due to the higher quantities of target DNA in the eDNA samples, resulting from the abundance of larvae and the potential for stable DNA to accumulate in stagnant water bodies [30,56], relative to an individual larval specimen. This is consistent with a prior study that demonstrates the feasibility of using similar molecular methods to monitor the introduced mosquito populations [30].

Sensitivity could not be calculated for eDNA samples categorized as No Detection because the calculation requires the presence of true positives. In the absence of true positives, the equation becomes mathematically undefined. The specificity for the No Detection-designated group was 75%, reflecting the proportion of correctly identified negatives. Within this group, the mean Ct value for false positive amplifications was 34.100 ± 4.903 for the *sierr*-PRB probe and 37.544 ± 1.348 for the *aegypti*-PRB probe. Non-specific amplification in the *AegySierr.*ID-qPCR assay was also observed in eDNA samples. For false positives within the *Aedes sierrensis*-designated group, or samples that had a high probability to be in the *Ae. sierrensis* class by LCA but were amplified by the *aegypti*-PRB probe, the Ct value was high (37.008 ± 1.732). Similarly, false positives within the *Aedes aegypti*-designated group showed *sierr*-PRB probe amplification with a high mean Ct (35.290 ± 3.133). Overall, Ct values associated with false positives clustered near or beyond the standard detection thresholds of qPCR assays, suggesting low-level, possibly nonspecific amplification or minor contamination. In cases where a sample’s Ct value exceeded the upper bound of the 95% CI established for true positives (i.e., mean Ct + CI), further scrutiny may be warranted. These samples may benefit from additional filtering, re-extraction, and qPCR testing, followed by classification using LCA to reduce the risk of misclassification due to non-specific amplification.

**Figure 9:**
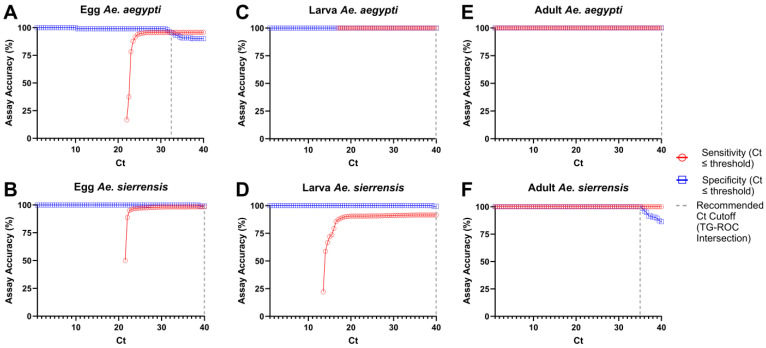
TG-ROC graphical analysis for determining Ct cutoffs for species classification using the *AegySierr.*ID-qPCR assay. Panels show assay performance by life stage: (**A**) *Ae. aegypti* egg tissue (n = 119); (**B**) *Ae. sierrensis* egg tissue (n = 114); (**C**) *Ae. aegypti* larva tissue (n = 31); (**D**) *Ae. sierrensis* larva tissue (n = 156); (**E**) *Ae. aegypti* adult tissue (n = 72); and (**F**) *Ae. sierrensis* adult tissue (n = 87). Sensitivity (red) and specificity (blue) curves are plotted against Ct values. Sensitivity (red) and specificity (blue) curves were plotted against Ct values. The dashed vertical line indicates the Ct cutoff, corresponding to the intersection of the two curves where applicable. When no clear intersection occurs or the curves remain overlapping, a default cutoff of Ct = 40 (the final qPCR cycle) was applied.

A TG-ROC analysis was used as an alternative method to establish species- and tissue-specific Ct thresholds for determining whether an unknown specimen is *Ae. aegypti* or *Ae. sierrensis*. Ct values that were previously determined for egg, larval, and adult tissue specimens that were confirmed as *Ae. aegypti* or *Ae. sierrensis* (Figure 6) were used for the TG-ROC analysis. As described in the Methods section, the TG-ROC decision rules yielded three classification outcomes. For *Ae. aegypti* eggs, the sensitivity and specificity curves intersected at Ct 32 to define the threshold for determining the species identity of egg samples (Figure 9). For adult *Ae. sierrensis* samples, the two amplification curves overlapped until Ct 35, after which they diverged, marking the only point of separation. Therefore, a Ct value of 35 was selected as the Ct cutoff for *Ae. sierrensis* adults (Figure 9). For all other sample types, the sensitivity and specificity curves either did not intersect or coincided across the entire range of Ct values, thereby setting the Ct threshold at 40 (Figure 9).

## 4. Conclusions

The *AegySierr.*ID-qPCR assay was designed to differentiate between *Ae. aegypti* and *Ae. sierrensis*, with the primary aim of helping vector control personnel avoid misidentifying *Ae. sierrensis* as *Ae. aegypti,* a critical distinction since *Ae. aegypti* is a vector of human pathogens such as dengue virus and requires species-specific control strategies to limit its spread. The assay demonstrated a high accuracy for the egg, larva, and adult stages, as well as for eDNA in water samples, including those collected in the absence of physical specimens.

This assay may be useful for the passive surveillance of *Ae. aegypti* by testing water in containers for eDNA in areas where the species is suspected. It could also support routine monitoring through oviposition trap networks, where water samples are periodically tested for mosquito eDNA to detect *Ae. aegypti* prior to the observation of physical specimens. Additionally, the assay may complement or serve as an alternative to morphological identification when specimens are damaged or in developmental stages that are difficult to distinguish visually.

In regions where a greater diversity of container- or treehole-breeding mosquito species are present, the assay should be applied with caution. Supplementary assessments, such as collecting specimens for morphological confirmation, should be considered in order to ensure accurate species identification. Additional validation with wild-collected samples from different geographic areas will be important for evaluating the assay’s robustness under diverse field conditions. The assay’s sensitivity and specificity should also be assessed against other sympatric *Aedes* species to assess its performance when additional container-breeding taxa are present. However, surveillance data collected by California vector control agencies from 2020 to 2024 show that *Ae. aegypti* accounted for 98.9% of over 1.1 million non-native container-breeding *Aedes* mosquitoes collected statewide, while *Ae. albopictus* and *Ae. notoscriptus* comprised 0.8% and 0.3%, respectively, and *Ae. japonicus* was not detected [57]. These patterns suggest that *Ae. aegypti*, the non-native target of the *AegySierr.*ID-qPCR assay, represents the majority of invasive container-breeding *Aedes* mosquitoes in regions where the native *Ae. sierrensis* also occurs.

## Figures and Tables

**Table 1:** Number of mosquitoes tested by species, life stage, and collection location.

Life Stage			
	Field *Ae. sierrensis*	Colony *Ae. aegy*-MV	Colony *Ae. sierr*-C
Egg	0	119	114
4th Instar Larva	132	31	24
Adult	47	72	40

**Table 2:** Primer and probe sequences that were developed to differentiate between *Ae. aegypti* and *Ae. sierrensis* in the *AegySierr.*ID-qPCR assay.

Name	Sequence (5′ → 3′)
Primers	
*aegypti*-F	TGATTAGCAACTTTACACGGAAC
*aegypti*-R	AGCTAATACTACTCCTGTTAAACCT
*sierrensis*-F	CCTCCTTCATTAACCCTACTACTTT
*sierrensis*-R	GATGATACTCCAGCTAAATGAAGAG
Probes	
*aegypti*-PRB	ABY-TCCAGCCCTTCTATGATCATTAGGATCTGT-IAbRQSp
*sierrensis*-PRB	FAM-TGGAGCTGG/ZEN/TACAGGATGAACTGT-IABkFQ

**Table 3:** Proportion of False Positives for each tested by location, species, and life stage.

Life Stage	Egg			Larva			Adult	
Sample	*Ae. sierr*-C	*Ae. aegy*-MV	*Ae. sierr*-C	Field-Collected *Ae. sierrensis*	*Ae. aegy*-MV	*Ae. sierr-C*	Field-Collected *Ae. sierrensis*	*Ae. aegy*-MV
**Proportion of False Positives (%)**	0.9%	10.0%	0.0%	0.7%	0.0%	0.0%	25.0%	8.3%
**Proportion of False Negatives (%)**	1.7%	4.2%	0.0%	11.8%	0.0%	0.0%	0.0%	0.0%

**Table 4:** Combined mean ± SD of Ct values for tissue samples by life stage and location.

Life Stage	Sample	Mean Ct	SD
Egg	*Ae. sierr-C*	24.819	3.117
	*Ae. aegy-MV*	24.533	2.314
Larva	*Ae. sierr-C*	17.725	1.244
	Field-collected *Ae. sierrensis*	17.576	4.940
	*Ae. aegy-MV*	19.713	1.594
Adult	*Ae. sierr-C*	14.259	0.881
	*Ae. sierr-C*	14.567	1.231
	*Ae. aegy-MV*	16.684	1.033

## Data Availability

The original contributions presented in this study are included in the article/Supplementary Material. Further inquiries can be directed to the corresponding author.

## Supplementary Materials

The following supporting information can be downloaded at: https://www.mdpi.com/article/10.3390/tropicalmed10080230/s1, Figure S1: A single instance of the false positive amplification for an *Ae. aegypti* sample that produced a Ct value with a ∆Rn below 1; Figure S2: Amplification plots for each *Ae. sierrensis* larval sample collected from Sonoma County, showing results for both *sierr*-PRB (blue) and *aegypti*-PRB (red) probes; Figure S3: Amplification plots for each *Ae. sierrensis* larval sample collected from Alameda County, showing results for both *sierr*-PRB (blue) and *aegypti*-PRB (red) probes; Figure S4: Amplification plots for each *Ae. aegypti* larval sample collected from Orange County, showing results for both *sierr*-PRB (blue) and *aegypti*-PRB (red) probes; Figure S5: Amplification plots for each *Ae. aegypti* adult sample collected from Orange County, showing results for both *sierr*-PRB (blue) and *aegypti*-PRB (red) probes; Figure S6: Amplification plots for all *Ae. sierrensis* eDNA samples displaying both *sierr*-PRB (blue) and *aegypti*-PRB (red) probes; Figure S7: Amplification plots for all *Ae. aegypti* eDNA samples displaying both *sierr*-PRB (blue) and *aegypti*-PRB (red) probes; File S1: Pooled standard deviation and mean equations.
