# Supplementary material for: Barcoding Quantitative PCR Assay to Distinguish Between Aedes aegypti and Aedes sierrensis
Source: Trop Med Infect Dis. 2025 Aug 15;10(8):230. doi: 10.3390/tropicalmed10080230 (PMC12390609; doi:10.3390/tropicalmed10080230)
Supplement: Supplementary file 1 [file tropicalmed-10-00230-s001.zip › S1 File.pdf]

Consolidation of Combined Mean and Standard Deviation Values

(1) $\sum x = mean \times n$

(2) $\sum x^2 = SD^2(n-1) + \frac{(\sum x)^2}{n}$

(3) $tn = sum\ of\ all\ (n)$

(4) $tx = sum\ of\ all\ \sum x$

(5) $txx = sum\ of\ all\ \sum x^2$

(6) $Combined\ mean = tx/tn$

(7) $Combined\ SD = \sqrt{\frac{txx - \frac{tx^2}{tn}}{tn-1}}$

Altman, D. G. Statistics with Confidence : Confidence Intervals and Statistical Guidelines; Bmj Books: London, 2011; pp. 28–31.
